# Analyzing lung cancer risks in patients with impaired pulmonary function through characterization of gut microbiome and metabolites

**DOI:** 10.1186/s12890-023-02825-6

**Published:** 2024-01-02

**Authors:** Jiahui Luan, Fuxin Zhang, Lijun Suo, Wei Zhang, Yige Li, Xiaofeng Yu, Bo Liu, Hongyun Cao

**Affiliations:** 1 Department of Clinical Microbiology, Zibo City Key Laboratory of Respiratory Infection and Clinical Microbiology, Zibo City Engineering Technology Research Center of Etiology Molecular Diagnosis, Zibo Municipal Hospital, Zibo, 255400 China; 2 Department of Pulmonary and Critical Care Medicine, Zibo Municipal Hospital, Zibo, 255400 China; 3 Department of General Thoracic Surgery, Zibo Municipal Hospital, Zibo, 255400 China; 4 Shandong University-Zibo Municipal Hospital Research Center of Human Microbiome and Health, Zibo, 255400 China; 5 State Key Laboratory of Microbial Technology, Shandong University, Qingdao, 266237 China

**Keywords:** Lung cancer, Gut microbiota, Metabolomics, Pulmonary function, Biomarkers

## Abstract

**Background:**

Lung cancer (LC) is one of the most devastating diseases worldwide, there is growing studies confirm the role of impaired lung function in LC susceptibility. Moreover, gut microbiota dysbiosis is associated with LC severity. Whether alterations in gut microbiota and metabolites are associated with long-term lung dysfunction in LC patients remain unclear. Our study aimed to analyze the risk factors in LC patients with impaired pulmonary function based on the characteristics of the gut microbiome and metabolites.

**Methods:**

Fecal samples from 55 LC patients and 28 benign pulmonary nodules patients were collected. Pulmonary ventilation function was graded according to the American Thoracic Society/ European Respiratory Society (ATS/ERS) method. LC patients were divided into 3 groups, including 20 patients with normal lung ventilation, 23 patients with mild pulmonary ventilation dysfunction and 12 patients with moderate or above pulmonary ventilation dysfunction. The fecal samples were analyzed using 16 S rRNA gene amplicon sequencing and metabolomics.

**Results:**

The gut microbiome composition between LC patients and benign pulmonary nodules patients presented clearly differences based on Partial Least Squares Discriminant Analysis (PLS-DA). Pulmonary ventilation function was positively correlated with LC tumor stage, the richness and diversity of the gut microbiota in LC patients with moderate or above pulmonary ventilation dysfunction increased significantly, characterized by increased abundance of *Subdoligranulum* and *Romboutsia*. The metabolomics analysis revealed 69 differential metabolites, which were mainly enriched in beta-Alanine metabolism, styrene degradation and pyrimidine metabolism pathway. The area under the curve (AUC) combining the gut microbiome and metabolites was 90% (95% CI: 79-100%), indicating that the two species and four metabolites might regarded as biomarkers to assess the prediction of LC patients with impaired pulmonary function.

**Conclusions:**

Our results showed that microbiome and metabolomics analyses provide important candidate to be used as clinically diagnostic biomarkers and therapeutic targets related to lung cancer with impaired pulmonary function.

**Supplementary Information:**

The online version contains supplementary material available at 10.1186/s12890-023-02825-6.

## Introduction

Lung cancer ( LC ) is one of the most common and the leading cause of cancer deaths worldwide [1]. Genetic susceptibility, gut microbiome and smoking are hypothesized to increase the risks of LC by shaping the tumor microenvironment and promoting the tumorigenesis [2]. Gut microbiota can influence the immune status of the host, which in turn increases susceptibility to malignancy. The gut microbiota and metabolites can enter the blood through the intestinal barrier, leading to a chronic inflammatory state in the organism [3]. Dysfunction of gut microbiota is believed to be associated with the occurrence and development of cancers, and studies have identified potential fecal biomarkers, satisfactory performances of these markers have been shown for diagnosing pancreatic cancer (AUC = 0.78–0.94) [4, 5], hepatocellular carcinoma (AUC = 0.8064) [6], lung adenocarcinoma (AUC = 0.76–0.976) [7, 8] and so on. Emerging studies have indicated that significant changes in the composition and function of the gut microbiota in patients with pulmonary diseases compared to healthy individuals [7, 9, 10]. The detection of differences in gut microbial communities between healthy individuals and LC patients could be used as a predictive tool for LC progression [11, 12]. Research on the role and mechanisms of intestinal flora and its metabolites in LC is beginning to receive widespread attention.

In recent years, many studies focus on the relationship between lung function and lung health. Kachuri et al. found that immune-mediated genetic pathways led to impaired lung function, with reduced FEV1 increasing the risk of squamous cell carcinoma and reduced FEV1/FVC increasing the risk of adenocarcinoma [13]. A large observational literature has found an increased risk of LC in patients with pulmonary insufficiency [14], but the possible relationship between LC and respiratory dysfunction has not been established. Li et al. revealed that the diversity of pulmonary microbiota in chronic obstructive pulmonary disease (COPD) patients with impaired lung function was similar [15]. *Airway lactobacilli* has the effects in ameliorates lung function decline [16]. Notably, patients with irritable bowel syndrome (IBS) with intestinal flora imbalance are prone to developed impaired lung function and chronic lung disease [17]. However, whether alterations in gut microbiota and metabolites are associated with long-term lung dysfunction in LC patients remain unclear.

Therefore, fecal samples were collected from 28 benign diseases patients and 55 LC patients, and further analyzed by 16 S rRNA amplicon sequencing and metabolomics to assess the diversity and structure of microbiota and differential metabolites in the fecal samples. Then, we graded lung cancer patients according to the American Thoracic Society/ European Respiratory Society (ATS/ERS) five level classification method for pulmonary ventilation impairment [18]. Interestingly, we found a positive correlation between tumor stage and pulmonary ventilation function, thus this study further analyzed the risk factors in LC patients with impaired pulmonary function based on the characteristics of the gut microbiome and metabolites.

## Methods

### Patients recruitment

Fecal samples from 82 LC patients and 36 patients with benign pulmonary diseases were collected from Zibo Municipal Hospital. The enrolled patients in this study were patients with suspicious nodules on CT images. Based on the pathological diagnosis results, 55 patients who were pathologically diagnosed with lung cancer and 28 patients who were diagnosed with benign pulmonary nodules diseases eventually met the inclusion criteria and were included in the final study. Performed pulmonary function tests on all enrolled patients, evaluated their lung ventilation and diffusion function using the Jeager MasterScreen instrument and detected changes in obstruction, restriction, and mixed pulmonary ventilation disorders. Further, pulmonary function was graded according to ATS/ERS method and ventilation parameters (FVC, FEV1, FEV1/FVC), LC patients were classified into 20 patients with normal pulmonary function (ZC group), 23 patients with mild pulmonary dysfunction (QD group) and 12 patients with moderate or above pulmonary ventilation dysfunction (ZZD group). Clinical data including age, gender, Body Mass Index (BMI), glucose, White Blood Cell (WBC), smoking history, tumor stage, tumor type, hemoglobin oxygen saturation and pulmonary ventilation function were assessed from hospital electronic medical records. The tumor stage of LC patients were diagnosed according to their pathological features using tumor node metastasis (TNM) scale classification of malignant tumors, patients were classified into four distinct disease stages (from I to IV).

All enrolled patients met the following criteria: [1] ≥ 18 but < 80 years old; [2] have been pathologically confirmed with lung cancer [3]. have been pathologically confirmed with benign pulmonary diseases [4]. each group patients have received antibiotics, corticoids, probiotics, prebiotics in the past 3 months were excluded; [5] with lung infection, pulmonary fibrosis, inflammatory bowel disease (IBD) and irritable bowel syndrome (IBS) were excluded; [6] hypertension, diabetes and previous airway surgery were excluded from this study. This study was approved by Ethics Committee of Zibo Municipal Hospital (Ethics No.20,220,311) and informed consents were obtained from all patients.

### Samples collection

All participants collected faces once before treatment, the collection of fecal samples from subjects were collected in the morning after an overnight fast. Discard the surface of the feces, collected the internal faces in sterile containers and evenly divided them into two parts on dry ice, which were used for 16 S rRNA genes sequencing and Non-targeted metabolomics, respectively. Subsequently, the samples were placed in anaerobic Bio-Bags and stored at -80 °C immediately.

### DNA extraction

Total fecal DNA was extracted using the OMEGA DNA kit, the integrity of the extracted DNA was checked using 1% agarose gel electrophoresis and DNA concentration and purity was determined using a Qubit 4 fluorometer.

### 16 S rRNA genes sequencing analysis

Specific primers with barcode were synthesized and the V3-V4 region of the 16 S rRNA gene was amplified using TransGen AP221-02. The PCR products were mixed and detected by 2% agarose gel electrophoresis. The products were purified by the AxyPrepDNA Gel Recovery Kit (AXYGEN, USA) and quantified using the QuantiFluor™ -ST Blue Fluorescence Quantification System (Promega, USA). Finally, sequencing was performed by Mejorbio Biopharmaceuticals on the llumina MiSeq platform (Illumina, USA).

UPARSE (version 7.0.1090 http://drive5.com/uparse/) was used for analysis of OTUs at 97% similarity. Alpha diversity indices (sobs, shannon, simpson, ace, chao, coverage) were calculated by Mothur (version 1.30.2 https://www.mothur.org/wiki/Download_mothur) to estimate community richness and diversity. The beta diversity of distance matrix was calculated by QIIME (version 1.9.1 http://qiime.org/install/index.html), the Non-metric multidimensional scaling (NMDS) analysis was performed by Vegan and differences between groups were analysed by Partial Least Squares Discriminant Analysis (PLS-DA). LEfSe analysis was performed to estimate differences of species abundance and the Kruskal-Wallis H test was used to assess the significance of the differences. Correlation coefficients of microbial communities were calculated using Spearman correlation algorithm and visualized by Cytoscape.

### Non-targeted metabolomics analysis

Metabolites were extracted from fecal samples, sequenced using a liquid chromatography-tandem mass spectrometry (LC-MS/MS) and the raw data were pre-processed using Progenesis QI (Waters Corporation, Milford, USA). PLS-DA was used to determine whether all samples could be clustered into different groups and KEGG pathway analysis [19] was used to identify signaling pathways with the enrichment of differential metabolites.

### Biomarkers identification and evaluation

The Receiver Operating Characteristic (ROC) curve analysis was employed to calculate the area under the curve (AUC) and the differential microbiota and metabolites with an AUC > 0.85 were considered as potential predictive markers.

### Statistical analysis

Patients clinical information was statistically analyzed using SPSS V.19.0, clinical characteristics were presented as mean ± standard deviation (SD) and Spearman rank correlation coefficients were used to assess the correlation between pulmonary function and tumor stage. For 16 S rRNA gene sequencing and Metabolome analysis, statistical calculations were performed using R3.4.3.

## Results

### Patients and clinical characteristics

In this study, a total of 55 lung cancer patients and 28 patients with benign pulmonary nodules diseases were enrolled. The clinical characteristics were showed in Table 1 and the information showed that there were no significant differences in gender, BMI, age, glucose, smoking, WBC and hemoglobin oxygen saturation. But pulmonary ventilation function was significant different in LC patients and benign pulmonary diseases patients (P = 0.016). Notably, pulmonary ventilation function was positively correlated with tumor stage (spearman rank correlation coefficient r = 0.286) and the correlation was statistically significant (Table 2).

**Table 1:** Clinical characteristics of the patients

	Lung Cancer patients (n = 55)	Benign diseases patients (n = 28)	P value
**Age yrs**			
mean ± SD	56.80 ± 17.39	57.71 ± 13.24	0.808
**Gender**			
Male, n (%)	28 (50.9%)	17 (60.7%)	0.397
Female, n (%)	27 (49.1%)	11(39.3%)	
**Body Mass Index (kg/m**^2^)			
mean ± SD	24.84 ± 2.94	25.51 ± 3.50	0.391
**Glucose**			
mean ± SD	5.15 ± 2.62	4.65 ± 1.06	0.225
**White Blood cell**			
mean ± SD	5.93 ± 1.45	6.72 ± 2.17	0.078
**hemoglobin oxygen saturation**			
mean ± SD	97.17 ± 1.24	96.93 ± 1.39	0.431
**Smoking status**			
Never smoking	44 (80%)	24 (85.7%)	0.183
Ever smoking	4 (7.3%)	0	
Current smoking	7 (12.7%)	4 (14.3%)	
**Pulmonary function**			
ZC ^(a)^	21 (38.2%)	15 (75%)	0.016*
QD ^(b)^	22 (40%)	3 (15%)	
ZZD ^(c)^	12 (21.8%)	2 (10%)	
**Tumor stage**			
Stage I, n (%)	43 (78.2%)		
Stage II, n (%)	7 (12.7%)		
Stage III, n (%)	3 (5.5%)		
Stage IV, n (%)	2 (3.6%)		
**Tumor type**			
Adenocarcinoma, n (%)	44 (84.6%)		
Squamous cell carcinoma, n (%)	6 (11.5%)		
small-cell carcinoma, n (%)	2 (3.8)		

^(a)^ Normal pulmonary function; ^(b)^ Mild pulmonary ventilation dysfunction; ^(c)^ moderate or above pulmonary ventilation dysfunction. *P < 0.05.

**Table 2:** Pulmonary ventilation function in relation to tumor staging

Pulmonary function	Stage I	Stage II	Stage III	Stage IV
ZC^(a)^	19(44.2%)	1(14.3%)	0(0.0%)	1(50%)
QD^(b)^	17(39.5%)	4(57.1%)	1(33.3%)	0(0.0%)
ZZD^(c)^	7(16.3%)	2(28.6%)	2(66.7%)	1(50%)
Total	43	7	3	2

^(a)^ Normal pulmonary function; ^(b)^ Mild pulmonary ventilation dysfunction; ^(c)^ moderate or above pulmonary ventilation dysfunction.

### Gut microbial profiles between Lung cancer patients and benign pulmonary Diseases patients

**Fig. 1:**
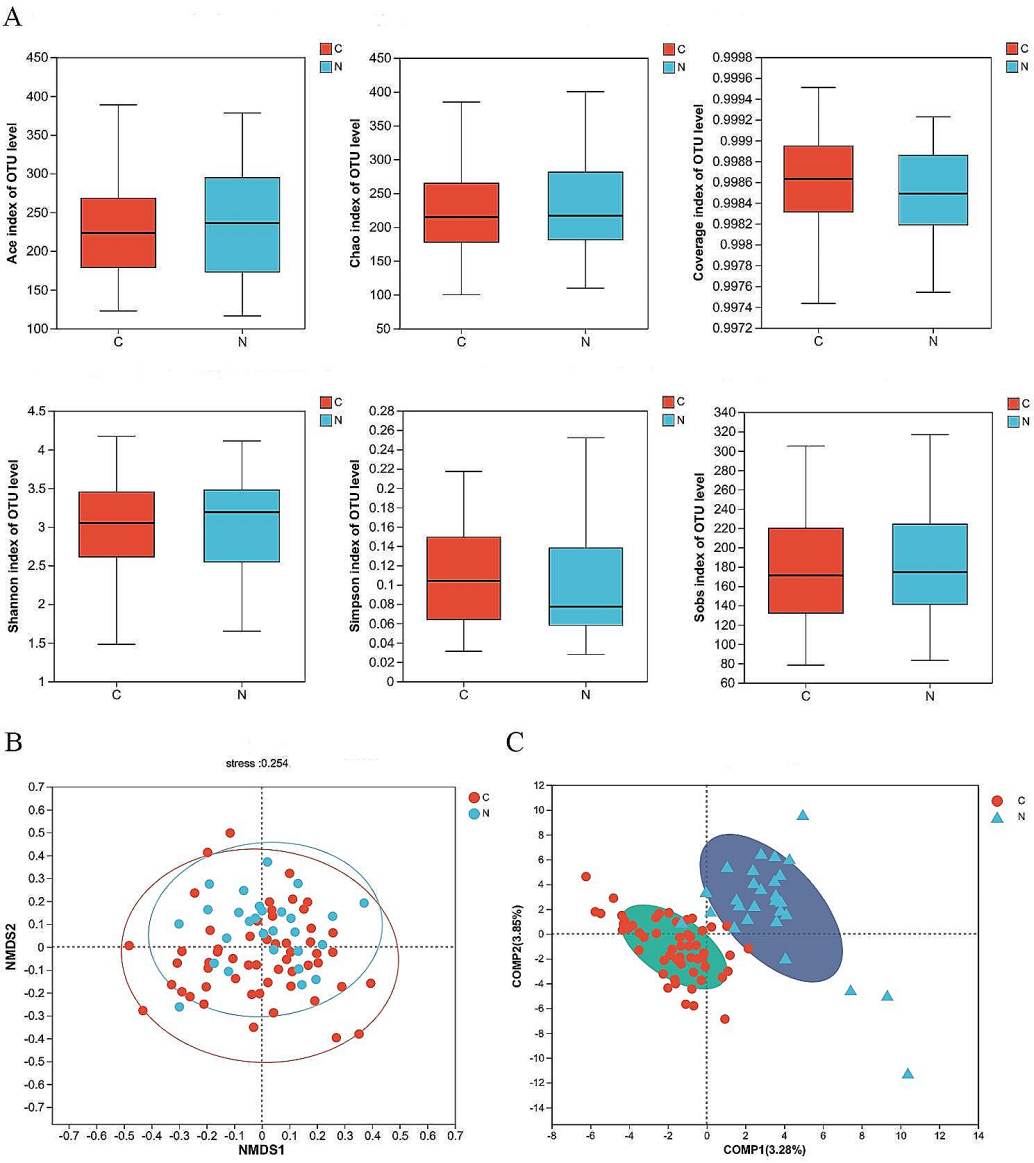
The diversity and richness of the gut microbiota in lung cancer patients (C) and benign diseases patients (N). **(A)** α-diversity between C and N groups based on the ace, chao, shannon, sobs, coverage and simpson indexes. **(B)** Non-metric multidimensional scaling (NMDS) analysis of C and N. **(C)** Partial Least Squares Discriminant Analysis (PLS-DA) of C and N

To explore the gut microbial profile between LC patients (C group) and benign diseases patients (N group), fecal samples were analyzed for 16s rRNA gene sequencing. OTU cumulative curves demonstrated that this sequencing was sufficient and credible (Fig. S1A, B). In the cohort, we discovered 1834 OTUs and the C group had 1008 exclusive OTUs (Fig. S1C). Besides, Ace, Chao, Coverage, Shannon, Simpson and Sobs indices were performed to compare the diversity and abundance of gut microbiome between the two groups. C group and N group had similar Alpha diversity (Fig. 1A). NMDS analysis showed a similar Beta diversity (Fig. 1B). However, the composition of the two groups presented clearly differences which were analyzed by PLS-DA (Fig. 1C).

### Gut microbial composition differences between Lung cancer patients and benign pulmonary Diseases patients

**Fig. 2:**
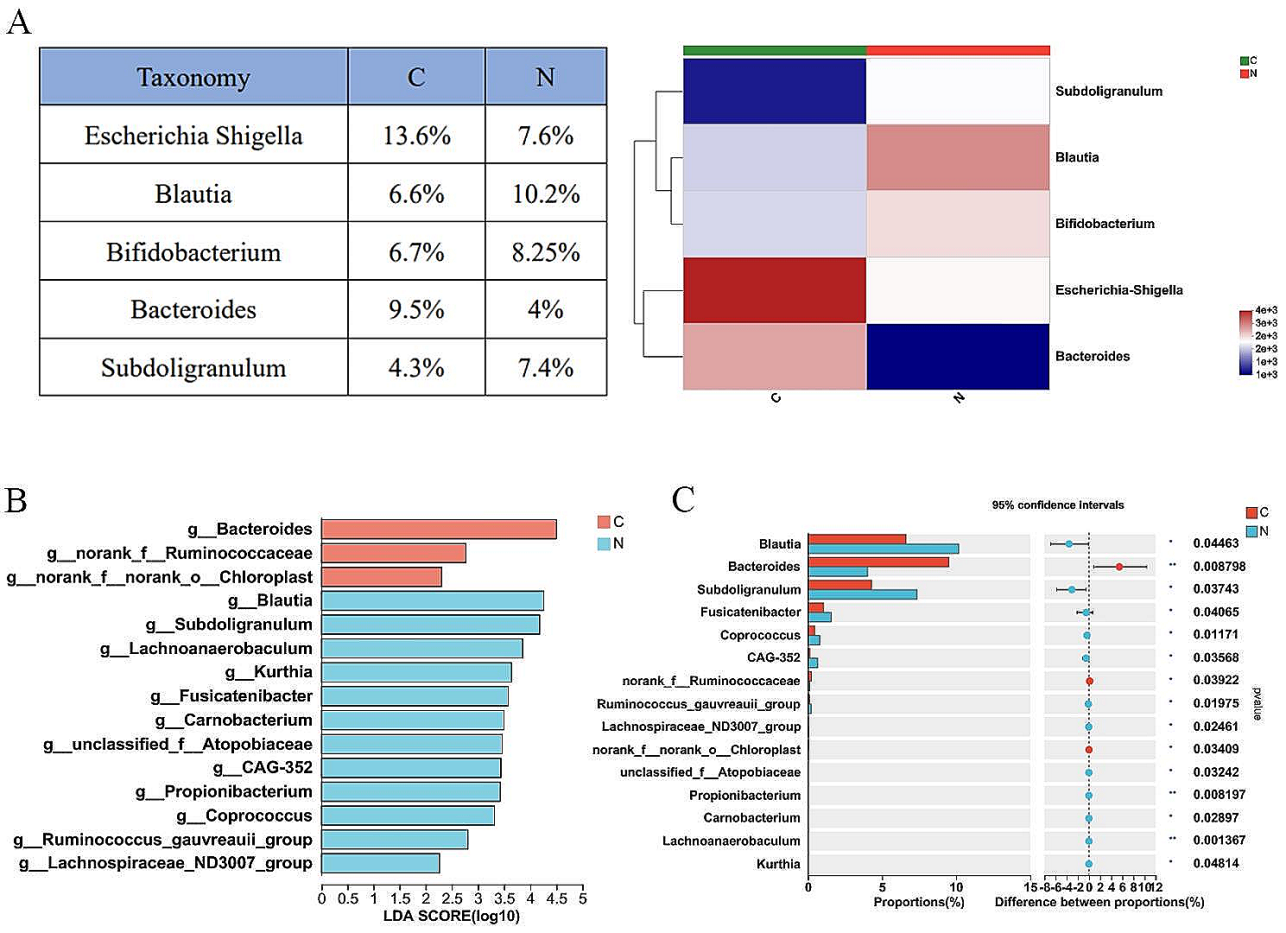
Gut microbial composition in lung cancer patients(C) and benign diseases patients(N). **(A)** Heatmap showing the distribution of the microbiota composition associated with C or N group. **(B)** Differential taxa at the genus level analyzed by linear discriminant analysis (LDA) scores (LDA > 2.0, P < 0.05). **(C)** Differential taxa at the genus level analyzed by Wilcoxon rank-sum test. *P < 0.05, **P < 0.01, ***P < 0.001

The analysis of species abundance of individual samples showed a different colony structure at genus level (Fig. 2A). Community heatmap analysis at genus level revealed that gut microbial composition differed between the two groups. In C group, *Eubacterium hallii group*, *Bacteroides* and *Bifidobacterium* were main genera. However, *Blautia*, *Bifidobacterium*, *Escherichia Shigella* and *Subdoligranulum* were four main genera in N group (Fig. 2B). LEfSe analysis revealed 21 genera with differential abundance in the two groups (LDA > 2.0, P < 0.05). Of these, *Blautia*, *Subdoligranulum* and *Fusicatenibacte* significantly enriched in the N group and *Bacteroides* enriched in the C group (Fig. 2C). Similarly, Wilcoxon rank-sum test showed that *Blautia* (P = 0.0446), *Subdoligranulum* (P = 0.0374) and *Fusicatenibacter* (P = 0.0406) were decreased significantly in C group, while *Bacteroides* (P = 0.0088) were clearly increased in C group.

### Gut microbial profiles of Lung cancer patients with impaired pulmonary function

**Fig. 3:**
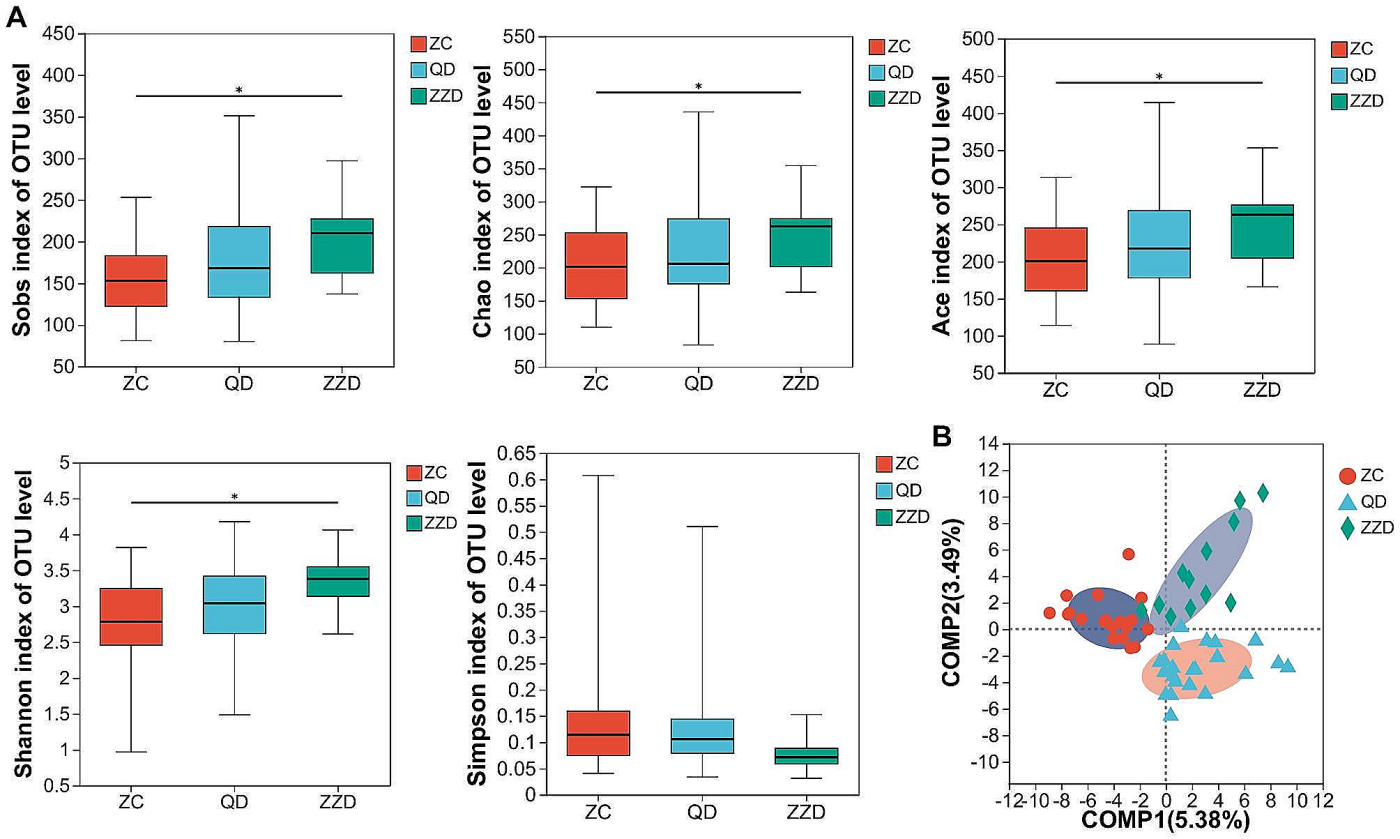
Gut microbial profiles in lung cancer patients with impaired pulmonary function. **(A)** α-diversity based on the Ace, Chao, Shannon, Simpson and Sobs indices. *P < 0.05. **(B)** Partial Least Squares Discriminant Analysis (PLS-DA) analysis between ZC, QD and ZZD patients. ZC, Normal pulmonary function; QD, Mild pulmonary ventilation dysfunction; ZZD, Moderate or above pulmonary ventilation dysfunction

AS pulmonary function was positively correlated with tumor stage. Next, we investigated the relationship of gut microbial profiles and lung cancer patients with impaired pulmonary function. Ace, Chao, Shannon, Simpson and Sobs indices were calculated to compare the diversity and abundance of gut microbiome among the LC patients with impaired pulmonary function. ZC group and QD group had a similar α-diversity, but the α-diversity of ZZD group was significantly increased (P < 0.05) (Fig. 3A). Analysis based on the PLS-DA model revealed that these groups could be significantly separated from each other (Fig. 3B).

### Metabolic and microbial profiling of gut microbiota in Lung cancer patients with impaired pulmonary function

**Fig. 4:**
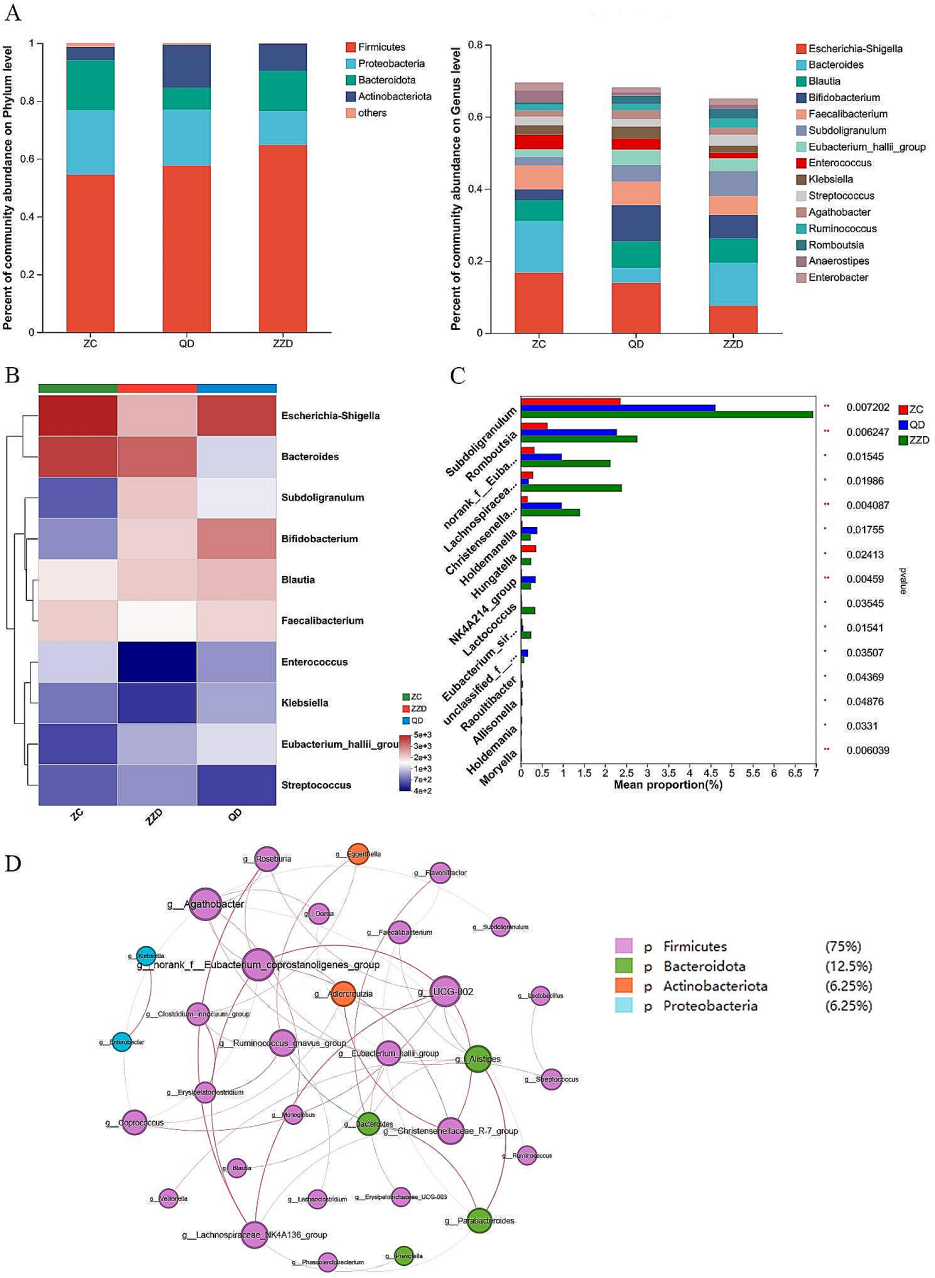
Gut microbial composition differences between ZC, QD and ZZD patients. **(A)** Relative abundance on phylum and genus levels. **(B)** Heatmap analysis on genus level. **(C)** 12 significantly different genera were showed by Kruskal wallis H test bar plot. **(D)** Correlation network of 50 different bacteria taxa from phylum to genus. *P < 0.05, **P < 0.01, ***P < 0.001

We analyzed the structure of the gut microbiota at different taxonomic levels. At the phylum level, *Firmicutes* was highly enriched in ZZD group, while *Proteobacteria* was enriched in ZC group. At the genus level, meaningful changes in the composition and abundance of gut microbiota could be observed between different groups (Fig. 4A). Community heatmap analysis at genus level revealed that gut microbial composition differed among the three groups, *Escherichia-Shigella* was the dominant genus, accounting for 16.8%, 14% and 7.7% of the ZC group, QD group and ZZD group, respectively. *Subdoligranulum* showed a opposite trend, accounting for 2.4%, 4.6% and 6.9% of the ZC group, QD group and ZZD group, respectively (Fig. 4B). Kruskal-Wallis H test showed that *Subdoligranulum* (P = 0.007), *Romboutsia* (P = 0.006) were decreased significantly in ZC group, while *Hungatella* (P = 0.024) were clearly increased in ZC group (Fig. 4C). Subsequently, Network analysis based on the measurement indexes (DC > 0.1, CC > 0.2, BC > 0.1) were used to identify the key microbiota (Fig. 4D). The result showed that *Christensenellaceae R-7 group* was negatively correlated with *Ruminococcus gnavus group*, *Eubacterium hallii Group* was positively correlated with *Blautia*, *Agathobacter* and *Dorea*.

In addition, fecal samples from 20 ZC patients, 23 QD patients and 12 ZZD patients were further analysed by LC-MS. A total of 6238 peaks were detected in the positive mode and 176 metabolites were annotated according to the KEGG database. In negative mode, 7274 peaks were detected and 102 were annotated according to the KEGG database. The data were normalized to verify the RSD values in the QC samples and the results showed good stability in both positive and negative modes (Fig. S2A). Veen diagram showed the number of specific and common metabolites (Fig. S2B). PLS-DA analysis revealed that the metabolites could be well separated among the three groups of specimens (Fig. S2C).

### Metabolomics profile changes and the predicted bacterial metabolic contribution in lung cancer patients with impaired pulmonary function

**Fig. 5:**
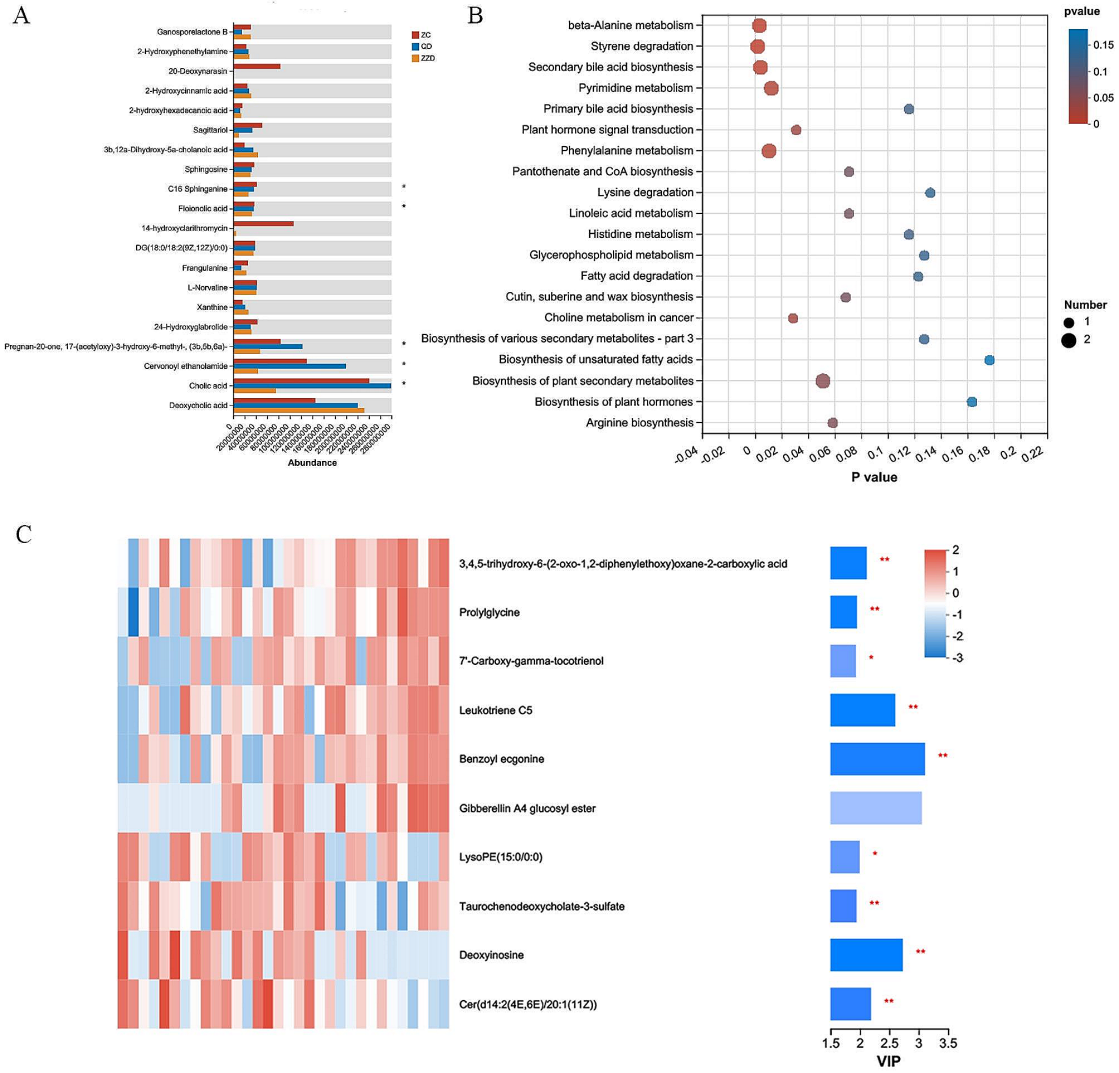
Differential metabolites and enriched signalling pathways. **(A)** 69 differential metabolites were filtered by Kruskal-Wallis H test. **(B)** KEGG pathway enrichment analysis. **(C)** Important metabolites displayed on variable importance in projection (VIP) plot obtained from OPLS-DA. *P < 0.05, **P < 0.01, ***P < 0.001

Kruskal-Wallis H test for differential metabolites among the three groups revealed a total of 69 differential metabolites, of which C16 Sphinganine, Floionolic acid, Cervonoyl ethanolamide and Cholic acid were most enriched in the ZC group (Fig. 5A). KEGG enrichment analysis revealed that the differential metabolites were mainly enriched in beta-Alanine metabolism, Styrene degradation, Secondary bile acid biosynthesis and Pyrimidine metabolism pathway (Fig. 5B). The metabolites that differed significantly between the ZC and ZZD groups were ranked according to the VIP values mapped by OPLS-DA (VIP ≥ 1) (Fig. 5C).

### Potential biomarkers for Lung cancer patients with impaired pulmonary function

**Fig. 6:**
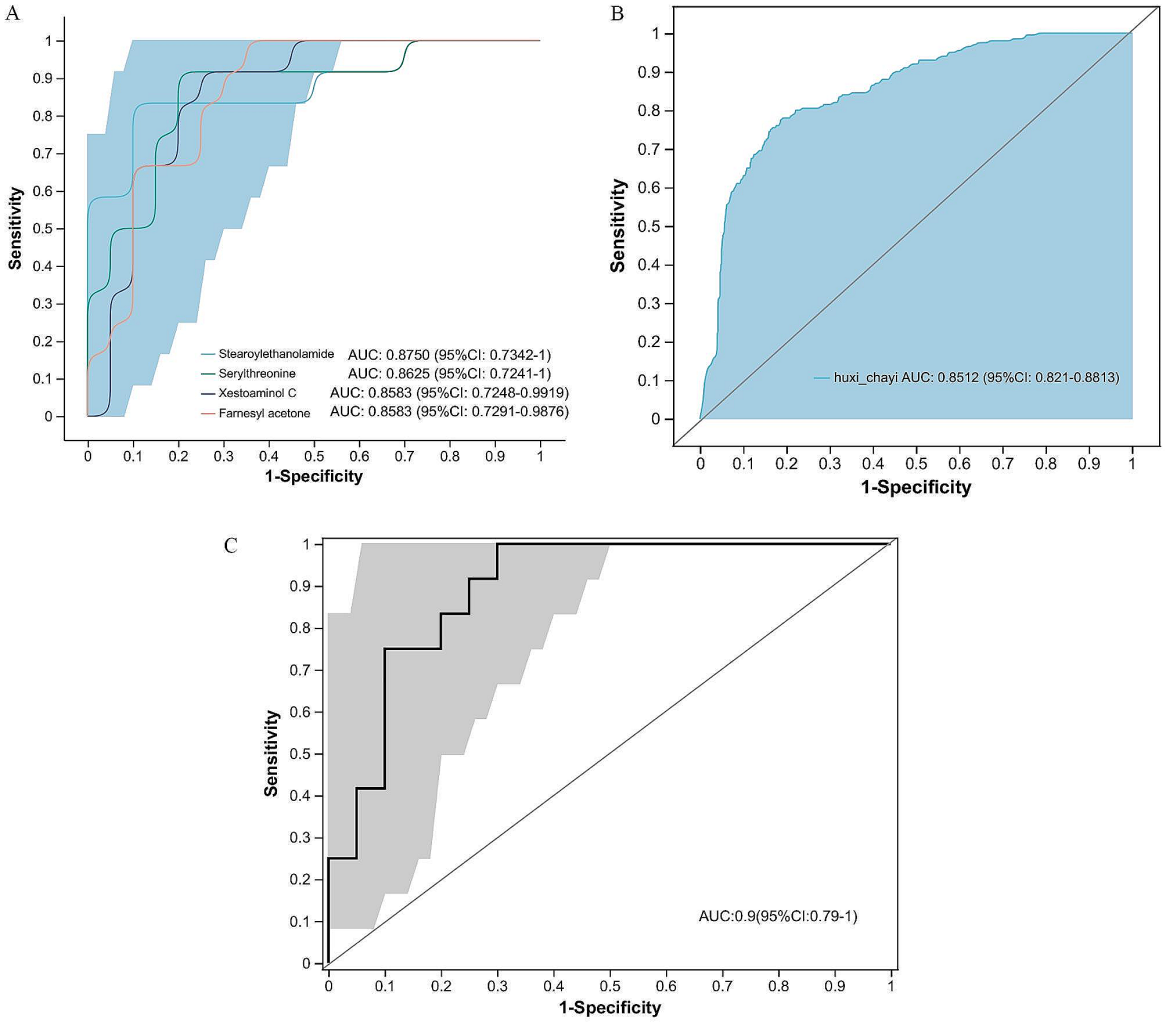
The random forest model based on the microbiota and metabolites to assess the candidate biomarkers. **(A)** Individual ROC curves for Stearoylethanolamide, Serylthreonine, Xestoaminol C and Farnesyl acetone. **(B)** ROC curves for the combination of the four metabolites. **(C)** ROC analysis of the combination of gut microbes and metabolites

Furthermore, ROC curves were performed to assess potential biomarkers in lung cancer patients with impaired pulmonary function. Metabolites with an AUC more than 0.85 were screened as potential biomarkers. As shown in the Fig. 6A, the AUC of Stearoylethanolamide, Serylthreonine, Xestoaminol C and Farnesyl acetone were 0.8750, 0.8625, 0.8583 and 0.8583. ROC analysis of the combination of the four metabolites showed comparable diagnostic power (AUC = 0.8512, Fig. 6B). However, the combination of remarkable 2 gut microbes and 4 metabolites showed a higher diagnostic power (AUC = 0.9) (Fig. 6C), indicated that the multi-dimensional data could better predict the risk of lung cancer progression with impaired pulmonary function.

## Discussion

Gut microbiota has been increasingly used as biomarkers for non-invasive disease diagnosis [20], and validated in many diseases, such as colorectal cancer [21], inflammatory bowel disease [22], non-small cell lung carcinomas [23] and liver cirrhosis [24]. In recent years, the relationship between gut microbiota and lung diseases has received great attention, irritable bowel syndrome (IBS) patients with dysbiosis of the gut microbiota are prone to developed impaired lung function [17], specific alterations in the composition and metabolism of the gut microbiota influence the occurrence and development of lung cancer [4, 10, 25]. In our study, changes in the composition and abundance of gut microbiota in LC patients were demonstrated, and possible candidates as markers for the diagnosis of LC patients impaired lung ventilation were identified.

The gut microbiota composition between LC patients and benign pulmonary diseases patients presented clearly differences based on PLS-DA, which is consistent with the finding by Zheng et al. and Zhuang et al. [4, 11]. Our study showed that *Blautia* and *Bifidobacterium* were more abundant in benign diseases patients and *Bacteroides* was more abundant in LC patients. *Blautia* is a major producer of butyrate [26, 27], which maintains gut environmental homeostasis and prevents inflammation by upregulating intestinal regulatory T cells and producing SCFAs [28, 29]. Moreover, Hosomi et al. demonstrated that *Blautia wexlerae* can induce intestinal metabolic changes produce anti-inflammatory effects, indicating that *Blautia* has effective effects on regulation of intestinal microecology [30]. Zhao et al. showed that *Bacteroides* and *Veillonella* were enriched in fecal samples of LC patients [31], which is in agreement with our studies. *Bacteroides fragilis* may provide virulence factors to sister cells by transferring virulence genes and these genes may contribute to the pathogenesis of extraintestinal organs [32]. To date, more and more evidences have shown that changes in gut microbiota and function can affect the effectiveness of anti-cancer treatment by regulating microbiota, such as probiotic interventions and fecal microbiota transplant (FMT) [33, 34].

During analyzing these patients clinical information, significant different pulmonary ventilation function in C and N patients (P = 0.016) was observed. Clinical and epidemiological studies have shown that patients with impaired lung function, especially those with COPD, are at higher risk of developing to lung cancer [35, 36]. Mendelian randomization analysis revealed histological-specific effects of reduced FEV1 and FEV1/FVC on LC susceptibility, suggesting that these indicators of impaired lung function may be pathogenic risk factors [13]. In our study, pulmonary ventilation function is positively correlated with tumor stage. Reduced FEV1 has been shown to increase the risk of squamous cell carcinoma and reduced the ratios of FEV1 to FVC increase the risk of adenocarcinoma and lung cancer in never smokers [13]. Gut microbiota is believed to play an important role in altering lung function [37]. However, no studies have investigated whether alterations in gut microbiota and metabolites are associated with long-term lung dysfunction in LC patients. Our study showed that ZC group and QD group had a similar α-diversity, but the α-diversity of ZZD group was significantly reduced (P < 0.05). In addition, these groups could be significantly separated from each other based on PLS-DA. Of which, *Hungatella* was significantly decreased in the ZZD group, suggesting a negative correlation with the aggravation of tumor stage in LC patients. However, *Subdoligranulum* and *Romboutsia* were significantly elevated in ZZD group. Chriswell et al. suggested that *subdoligranulum* stimulated joint swelling and inflammation [38]. Ni et al. demonstrated that *subdoligranulum* is the dominant biomarker that distinguish vitiligo patients from healthy controls [39]. In contrast, Lloyd-Price *at al*. showed that *subdoligranulum* was markedly increased in inflammatory bowel diseases (IBD), considering as a butyrate producer [40]. Due to the variability of findings, therefore further research is needed to demonstrate the role of *Subdoliganulum.* It has been reported that invasive mechanical ventilation leads to lung microbiota changes in rat models, which mainly characterization by the *Romboutsia* and *Tubriciactor* genera [41]. We speculate that the alterations of genera may be related to the patient dietary habits and a history of nasal oxygen therapy. So the role of these genera in LC patients with pulmonary dysfunction needs to be further investigated in a larger sample cohort.

Then, in the metabolomics analysis of fecal samples, bile acids were significantly decreased in ZZD group. Bile acid biosynthesis changed is a collaborative effect between the host and gut microbiome [42]. The reduction of secondary bile acids may alter the composition of gut microbiota and promote an intestinal inflammation profile [43]. Nie et al. revealed that bile acid metabolism is related to poor prognosis, and may potentiate migration of lung adenocarcinoma (LUAD) [44]. TGR5, the bile acid receptor, as a negative regulator of the NF-κB and AKT pathway, may effectively inhibit the progression of non-small cell lung cancer (NSCLC) [45].

The ROC curve analysis identified four potential biomarkers of diagnostic significance, Stearoylethanolamide, Serylthreonine, Xestoaminol C and Farnesyl acetone with AUC of 0.8750, 0.8625, 0.8583 and 0.8583, respectively. Stearoylethanolamide, an endogenous cannabinoid-like compound with pro-apoptotic activity, is found in the human brain to support the blood-brain barrier in acute systemic inflammation [46, 47]. Terpenoids related compounds exhibit high antibacterial activity against gram-negative bacteria and the very high cytotoxic activity profile of farnesol gives it potential as an anticancer agent [48]. ROC curve analysis between the gut microbes and metabolites showed that the diagnostic power was significantly increased (AUC = 0.9). Previous studies revealed that combining of the bacteria and the clinical tumor markers showed a higher ROC value for predicting LC [49]. Besides, the diagnostic power for predicting colorectal cancer was significantly increased based on the combination of metabolites and bacterial markers [50]. These studies suggesting that joint multi-dimensional data could be used to better predict disease risks. In many cases, gut flora structure has been correlated with the severity of respiratory diseases, such as NSCLC, COVID-19 and COPD [51–53].

In conclusion, the intestinal microecology of patients with LC and benign pulmonary diseases patients were characterized in this study. It is revealed that impaired lung ventilation may influence disease severity in LC patients and a predictive model could be developed based on the combination of gut microbes and metabolites for assessing the severity of lung cancer. In the future, a larger sample of the validation queue is needed to validate that alterations in gut microbiota and metabolites are associated with long-term lung dysfunction in LC patients.

## Conclusions

Our results showed that microbiome and metabolomics analyses provide important candidate to be used as clinically diagnostic biomarkers and therapeutic targets related to lung cancer with impaired pulmonary function.

### Electronic supplementary material

Below is the link to the electronic supplementary material.

Supplementary Material 1

## Data Availability

The datasets presented in this study can be found in online repositories. The names of the repository/repositories and accession number(s) can be found below: https://www.ncbi.nlm.nih.gov/, PRJNA956658.
