# Supplementary material for: Analyzing lung cancer risks in patients with impaired pulmonary function through characterization of gut microbiome and metabolites
Source: BMC Pulm Med. 2024 Jan 2;24:1. doi: 10.1186/s12890-023-02825-6 (PMC10759599; doi:10.1186/s12890-023-02825-6)
Supplement: Supplementary file 1 — Supplementary Material 1 [file 12890_2023_2825_MOESM1_ESM.docx]

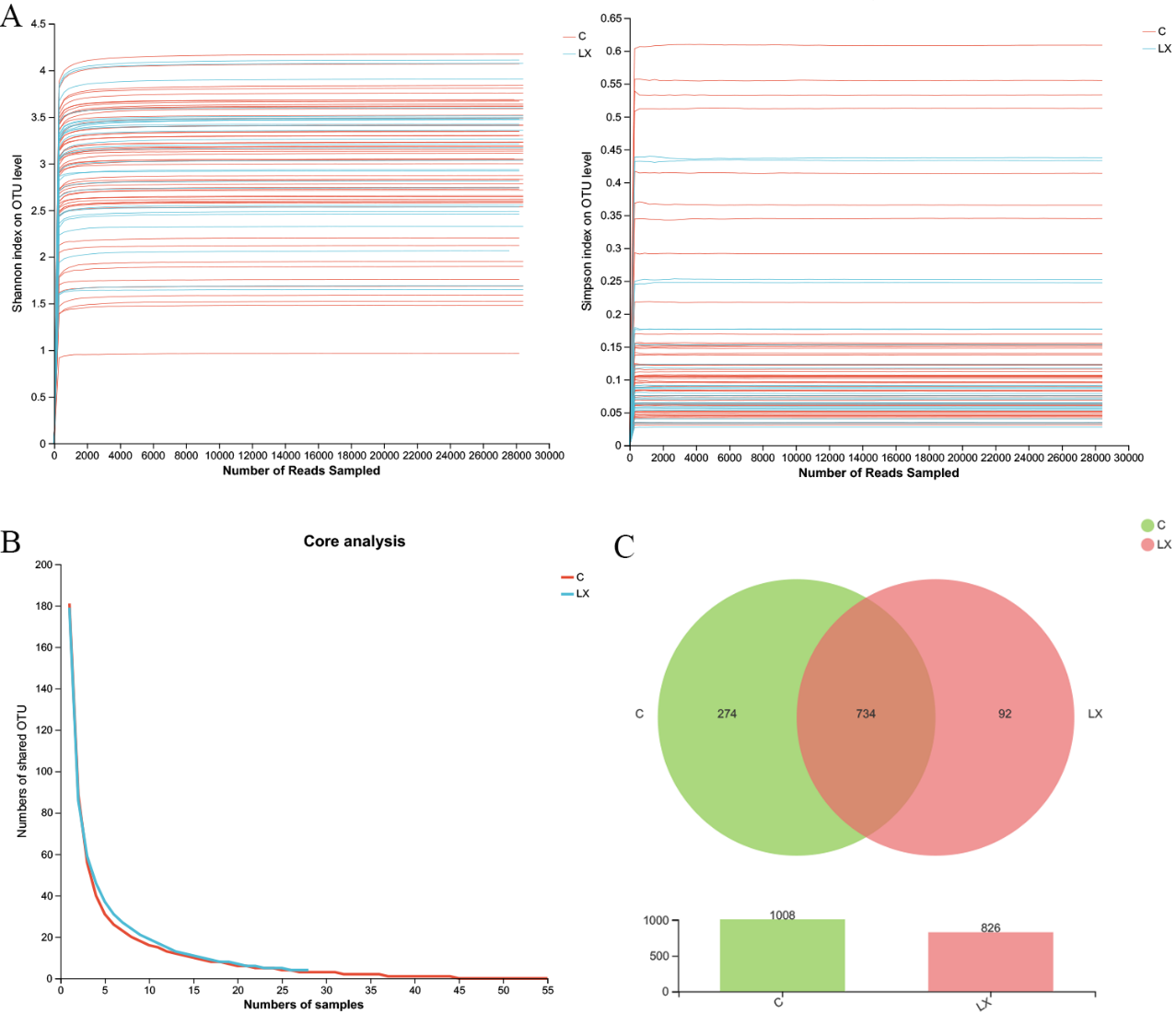


Figure S1 OTU clustering and Venn diagram between lung cancer patients (C) and benign diseases patients (N). (A) OTU cumulative curves. (B) Pan and Core curves. (C) Venn diagram.


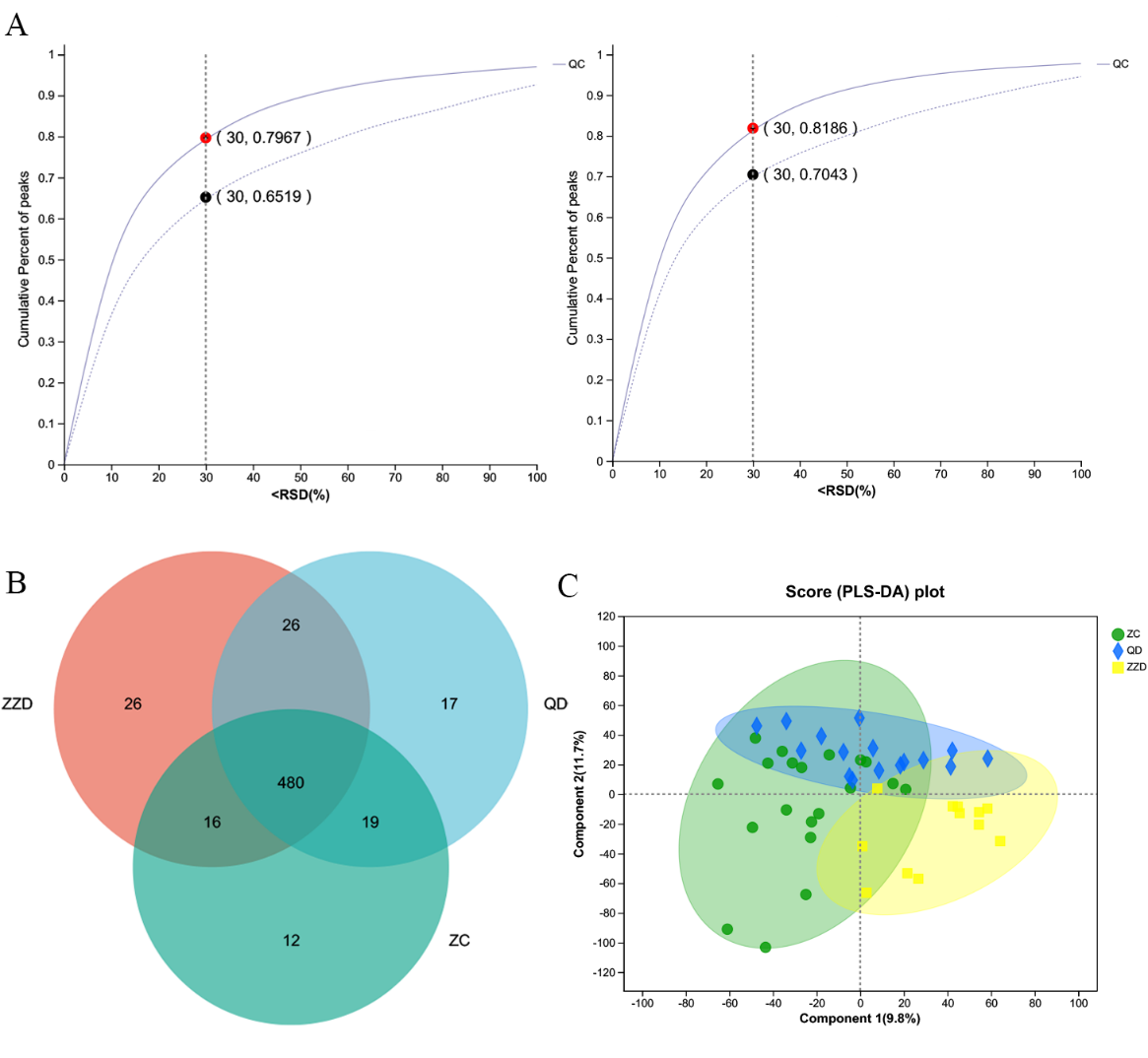


Figure S2 The stability of fecal samples was tested from LC-MS. (A) In the positive and negative mode. (B) Venn diagram. (C) OPLS-DA showed that metabolites were separated into three distinct clusters.
